# Adsorption and photocatalytic scavenging of 2-chlorophenol using carbon nitride-titania nanotubes based nanocomposite: Experimental data, kinetics and mechanism

**DOI:** 10.1016/j.dib.2020.106664

**Published:** 2020-12-16

**Authors:** M.A. Barakat, Rajeev Kumar, Jamiu O Eniola

**Affiliations:** a Department of Environmental Sciences, Faculty of Meteorology, Environment and Arid Land Agriculture, King Abdulaziz University, Jeddah 21589, Saudi Arabia; b Central Metallurgical R & D Institute, Helwan 11421, Cairo, Egypt

**Keywords:** C_3_N_4_/TiO_2_ nanotube, 2-Chlorophenol, Adsorption, Photocatalysis, Kinetics, Mechanism

## Abstract

Adsorption and interaction of pollutant species on surface of the catalyst materials play an important role on the photocatalysis process. Herein, experimental data on the adsorption behavior of 2-chlorophenol (2-CP) onto graphitic pure carbon nitride (C_3_N_4_), titania nanotubes (TiO_2_—NTs) and carbon nitride/titania nanotubes nanocomposite (C_3_N_4_/TiO_2_—NTs) from synthetic wastewater has been summarized. The data on photocatalytic degradation of the 2-CP under both ultraviolet (UV) and visible light irradiation is also presented. This work also evaluates the 2-CP scavenging efficiency of C_3_N_4_/TiO_2_—NTs nanocomposite prepared by calcination of 2 wt.% melamine with TiO_2_—NTs at 450 °C. The adsorption and photocatalysis experiments were conducted for 180 min at pH 7 with 100 mL solution of 2-CP (40 mg/L) and 0.05 g catalyst material. The acquired data can be valuable to identify the equilibrium time for 2-CP adsorption onto C_3_N_4_, TiO_2_—NTs, and C_3_N_4_/TiO_2_—NTs nanocomposite. Moreover, the obtained data can be useful to identify the suitable light source for the decomposition of 2-CP in the aquatic environment. The evaluated kinetic data might be significant for identifying the adsorption and photocatalysis reaction rate onto the applied catalyst materials. The obtained adsorption and photocatalysis data have been compared with that in literature to identify the adsorption and photocatalysis behavior of 2-CP on numerous catalysts at different experimental conditions.

## Specifications Table

**Table:** 

Subject	Environmental science, material science
Specific subject area	Wastewater purification, material synthesis, adsorption, photocatalysis,
Type of data	Tables, Figures
How data were acquired	The amount of 2-chlorophenol in the aqueous solution before and after adsorption and photocatalysis was analyzed by HACH DR6000 UV–visible spectrophotometer. UV-visible diffuse reflectance spectra of the C_3_N_4_, TiO_2_—NTs, and C_3_N_4_/TiO_2_—NTs nanocomposite were recorded on VARIAN Cary 500, USA.
Data format	Raw and analyzed
Parameters for data collection	At different times, the experimental data were obtained in the dark and under the UV and visible light illumination to analyze the adsorption and photocatalytic efficiency of the synthesized pure and hybrid material. The equilibrium attainment time for 2-CP adsorption was studied in the dark while the photocatalytic decomposition under UV (112 W) and visible light irradiation (104 W). Moreover, the efficiency of the C_3_N_4_, TiO_2_—NTs, and C_3_N_4_/TiO_2_—NTs nanocomposite for 2-CP adsorption and photocatalytic degradation were compared.
Description of data collection	The data related to adsorption and photocatalysis was collected in the form of the concentration of the 2-CP. A certain amount of 2-CP solution was drawn every 30 min and filtered by a 0.22 μm membrane syringe filter. The adsorption and photocatalysis experiments were performed between 0 and 180 min. The solution pH 7 was kept constant during the whole adsorption and photocatalysis process.
Data source location	King Abdulaziz University, Jeddah, Saudi Arabia
Data accessibility	Raw data are provided with the article in a supplementary file. Mendeley Data under identification number: https://data.mendeley.com/datasets/vwkrtdg85b/3
Related research article	M Anjum, R Kumar, SM Abdelbasir, MA Barakat. Carbon nitride/titania nanotubes composite for photocatalytic degradation of organics in water and sludge: Pre-treatment of sludge, anaerobic digestion and biogas production. Journal of environmental management 223, 2018, 495–502. https://doi.org/10.1016/j.jenvman.2018.06.043

## Value of the Data

• Data is valuable to develop new hybrid adsorbent and catalyst materials for the efficient removal of the contaminants from the wastewater.
• The adsorption data revealed that pure C_3_N_4_ is a better adsorbent than TiO_2_—NTs, and C_3_N_4_/TiO_2_—NTs nanocomposite for the removal of 2-CP.
• Kinetic data can be used to find the rate of 2-CP adsorption and photocatalytic degradation onto C_3_N_4_, TiO_2_—NTs, and C_3_N_4_/TiO_2_—NTs nanocomposite.
• Data could be valuable to identify a suitable radiation source for photocatalytic applications.
• Data may be applicable to find the equilibrium time for the adsorption and photocatalysis of 2-CP onto C_3_N_4_, TiO_2_—NTs, and C_3_N_4_/TiO_2_—NTs nanocomposite.
• Data could be used to identify the band gap energy, conduction band and valance band energy level of the C_3_N_4_, TiO_2_—NTs, and C_3_N_4_/TiO_2_—NTs nanocomposite.

## 1 Data Description

The data presented in the article explore the adsorption and photocatalytic properties of C_3_N_4_, TiO_2_—NTs, and C_3_N_4_/TiO_2_—NTs nanocomposite for scavenging of 2-CP from aqueous solution [1]. Adsorption plays a vital role in the photocatalysis process. It is assumed that good interaction between the pollutant species with the catalyst surface facilitates better photocatalytic decomposition [2], [3], [4]. Prior to starting the photocatalysis of the pollutant, adsorption was performed in the dark to identify the saturation of the catalyst and to determine the pollutant scavenging efficiency of the materials during adsorption and photocatalysis [5,6]. Adsorption kinetic analysis is important to find the rate of the 2-CP removal and to identify the nature of the process, i.e. chemisorption or physical sorption [7]. The liner plots and the adsorption kinetic parameters such as calculated adsorption capacity (q_e_), values of the rate constant (k) and correlation coefficient (R^2^) have been reported.

**Fig. 1:**
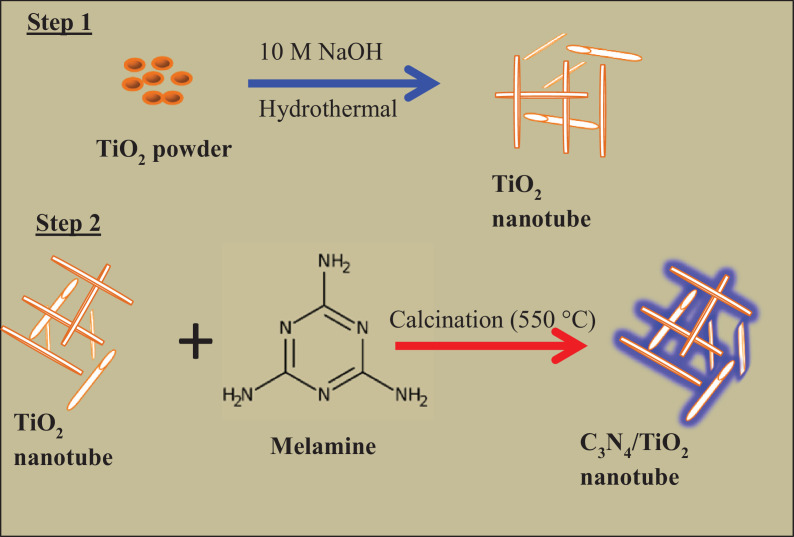
**The** schematic diagram for the synthesis of C_3_N_4_/TiO_2_—NTs nanocomposite.

**Fig. 2:**
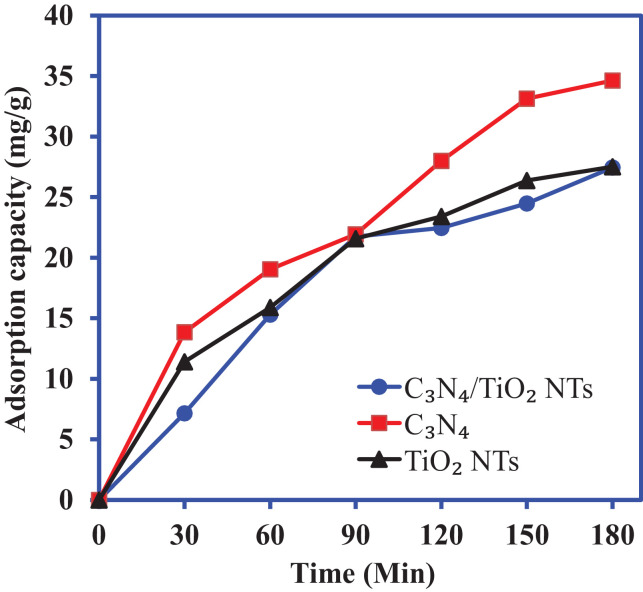
The adsorption of 2-CP onto C_3_N_4_, TiO_2_—NTs, and C_3_N_4_/TiO_2_—NTs nanocomposite.

**Fig. 3:**
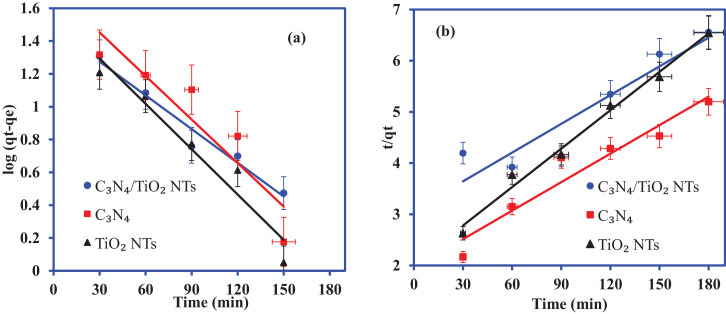
Plots for pseudo-first order and (a) pseudo-second order kinetic models for 2-CP adsorption onto C_3_N_4_, TiO_2_—NTs, and g-C_3_N_4_/TiO_2_—NTs nanocomposite.

Fig. 1 shows a schematic diagram for the synthesis of the C_3_N_4_/TiO_2_—NTs nanocomposite in two steps. The adsorption proprieties of C_3_N_4_, TiO_2_—NTs, and C_3_N_4_/TiO_2_—NTs nanocomposite for 2-CP scavenging is shown in Fig. 2. The liner plots for adsorption equilibrium data fitted to the pseudo-first order and pseudo-second order kinetic models are shown in Fig. 3 at 40 mg/L of 2-CP concentration. The values of the pseudo-first order and pseudo-seconder order kinetic parameters obtained from the liner plots in Fig. 3 are included in Table 1.

**Table 1:** The values of kinetic parameters for 2-CP adsorption onto C_3_N_4_/TiO₂ NTs, C_3_N_4_ and TiO₂-NT.

	Pseudo-first order			Pseudo-first order Pseudo-second order			
	qe(^exp)^ (mg/g)	R^2^	K_1_ (1/min)	qe^cal^	R^2^	K_2_ (g/mg/min)	qe^cal^ (mg/g)
C_3_N_4_/TiO₂ -NTs	27.45	0.9581	0.018	36.54	0.8664	0.00011	53.48
C_3_N_4_	34.62	0.914	0.002	57.59	0.9280	0.00018	53.76
TiO₂-NTs	27.5	0.9567	0.020	34.7	0.9888	0.00030	40.0

**Fig. 4:**
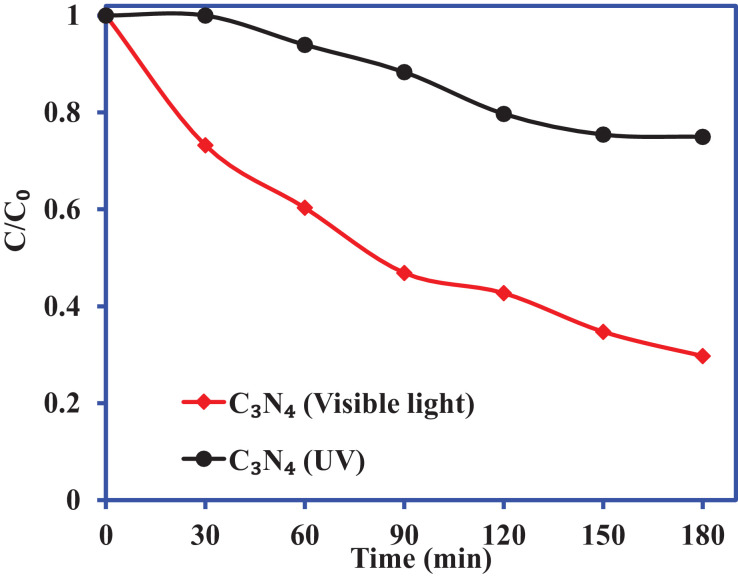
Photocatalytic degradation of 2-CP over C_3_N_4_ under UV and visible light irradiation.

**Fig. 5:**
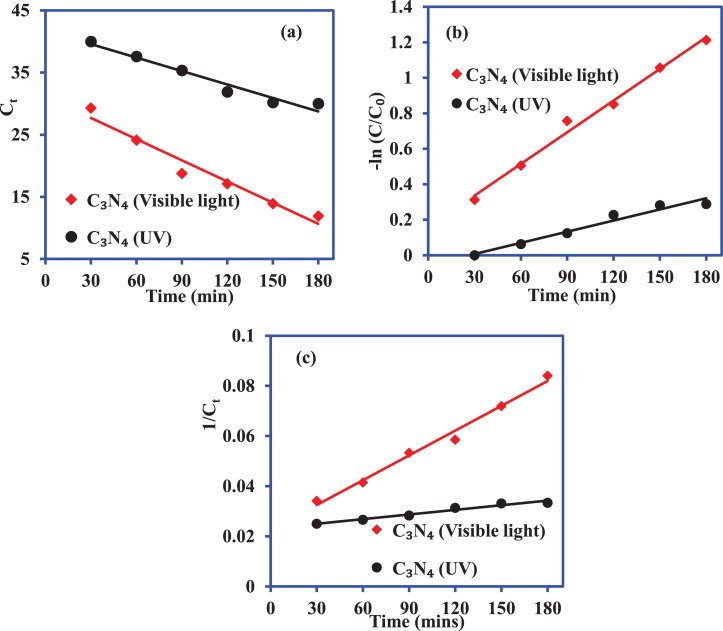
Kinetics plots of 2-CP degradation over C_3_N_4_ under UV and visible light irradiation (a) zero-order kinetic (b) first-order kinetic and (c) second-order kinetic models.

Fig. 4 shows the plot for the degradation of the 2-CP over C_3_N_4_ under UV (light intensity 112 W) and visible light irradiation (light intensity 104 W) for 180 min, and 2-CP concentration was 40 mg/L. Fig. 5 illustrates the liner plots for the zero-order, first-order, and second-order kinetic models. The values of zero-order, first-order, and second-order kinetic models are mentioned in Table 2.

**Table 2:** The values of kinetic parameters for the degradation of 2CP by C_3_N_4_ in the presence of UV and visible light.

Kinetic models	Parameters	C_3_N_4_ (Visible light)	C_3_N_4_ (UV)
Zero-order	k_0_ (mg/L. min)	113.6 × 10^−3^	72.1 × 10^−3^
	R^2^	0.9587	0.9562
First-order	k_1_ (1/min)	6 × 10^−3^	2.1 × 10^−3^
	R^2^	0.9902	0.9608
Second-order	k_2_ (L/mg. min)	3 × 10^−4^	6 × 10^−5^
	R^2^	0.9871	0.9633

**Fig. 6:**
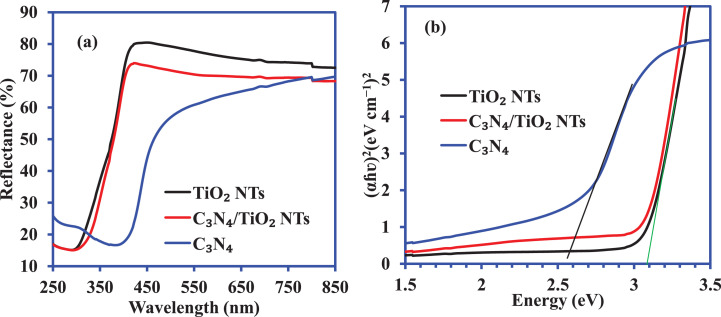
(a) UV-visible diffuse reflectance spectra and (b**)** Tauc plot for band gap energy calculation of C_3_N_4_, TiO_2_—NTs, and C_3_N_4_/TiO_2_—NTs nanocomposite.

**Fig. 7:**
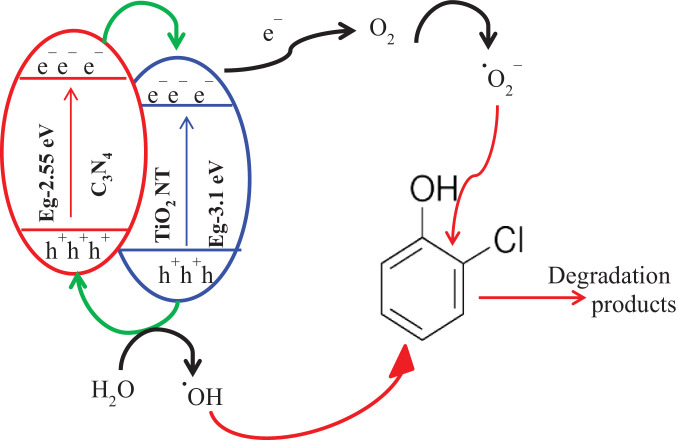
The schematic diagram of the photocatalysis mechanism of 2-CP degradation by C_3_N_4_/TiO_2_—NTs nanocomposite.

The UV-visible diffuse reflectance spectra of C_3_N_4_, TiO_2_—NTs, and C_3_N_4_/TiO_2_—NTs nanocomposite is shown in Fig. 6a. The band gap energy calculated using the Tauc plot is shown in Fig. 6b. A schematic diagram shown in Fig. 7 indicates the active radical species' production for the decomposition of 2-CP into the mineral by-products. A comparison of the adsorption capacity and the photocatalytic efficiencies of various adsorbents and catalysts for 2-CP scavenging are shown in Tables 3 and 4, respectively.

**Table 3:** Comparison of the adsorption efficiency of 2CP by different materials.

Material	Removal	Experimental conditions	Ref
Amberlite XAD-16 resin	2.27 mmol/g	pH-6, conc. – 11.68 mmoles/L	[8]
(TNTs/ACF)	59.9 (mg/g)	pH- 5, conc.- 20 mg/L, time – 15 min	[9]
ZnO/Clay	> 16%	pH-7, conc. – 20 mg/L, mass- 0.2 g	[10]
Clay	> 20%	pH-7, conc. – 20 mg/L, mass- 0.2 g,	[10]
ZnO	> 4%	pH-7, conc. – 20 mg/L, mass- 0.2 g,	[10]
C_3_N_4_/TiO_2_ -NTs	27.45 mg/g	pH-7, conc. – 40 mg/L, mass- 0.05 g	This work
TiO_2_—NTs	27.5 mg/g	pH-7, conc. – 40 mg/L, mass- 0.05 g	This work
C_3_N_4_	34.62 mg/g	pH-7, conc. – 40 mg/L, mass- 0.05 g	This work

**Table 4:** Comparison of photolysis and photocatalytic efficiencies of various materials used for the degradation of 2-CP.

Material	Degradation (%)	Experimental conditions	Ref
ZnO/Clay	88	pH-8.7, conc. – 20 mg/L, mass- 0.2 g	[10]
ZnO	61	pH-8.7, conc. – 20 mg/L, mass- 0.2 g	[10]
5% Ag-doped TiO2	74	pH −10.5, conc. – 50 mg/L, mass- 0.005 g	[11]
0.2% Ru/TiO_2_	53	pH – 6, conc. – 100 mg/L, mass- 0.002 g	[12]
Co-doped TiO_2_	93.4	pH – 9, conc. – 50 mg/L, mass- 0.01 g, time- 3 h	[13]
ZnO	55.6	pH – 9, conc. – 25 mg/L, time – 3 h	[14]
			
Photolysis	27	Time – 10 h	[15]
Photolysis	17.05	pH-7, conc. – 40 mg/L, time – 3.5 h, mass- 0.05 g	This work
C_3_N_4_ (UV)	25.02	pH-7, conc. – 40 mg/L, mass- 0.05 g	This work
C_3_N_4_ (visible light)	70.25	pH-7, conc. – 40 mg/L, mass- 0.05 g	This work

## 2 Materials and Methods

### 2.1 Materials

The 2-chlorophenol (2-CP) used as a model pollutant was supplied by Merck, Pvt Ltd. The powdered TiO_2_ used for the synthesis of TiO_2_ NT was provided by (P-25 Degussa Co.). C_3_N_4_ was synthesized by from melamine which was obtained from Sigma Aldrich.

### 2.2 Synthesis

Herein, thermal methods were used to synthesize the C_3_N_4_, TiO_2_ NTs, and C_3_N_4_/TiO_2_—NTs nanocomposite, as previously reported [1]. The C_3_N_4_/TiO_2_—NTs nanocomposite used in this study was synthesized by calcination of 2 wt% of melamine with TiO_2_ NTs.

### 2.3 Adsorption and photocatalysis experiment

Adsorptive removal of 2-CP was investigated using the batch adsorption process, and all the photocatalytic experiments were conducted in the UV/visible light photochemical reactor (Luzchem, LZC 4 V, Canada). The batch adsorption process and photocatalysis experiments were conducted in the 250 ml beaker containing the appropriate dose of catalyst/adsorbent (0.05 g) and 100 ml of 40 mg/L 2-CP solution. The pH of the 2-CP solution was adjusted to 7, using 0.1 M HCl or 0.1 M NaOH. The mixture containing catalyst was agitated at a speed of 200 rpm on a magnetic stirrer in the dark for adsorption experiments and in the presence of UV or visible light radiation and aeration for photocatalysis experiments. Samples were extracted at 30 mins intervals (30 – 180 min) using the pre-rinsed syringe and filtered through the 0.22 μm membrane filter. The concentration of 2-CP after adsorption and photocatalysis was analyzed using the UV–visible spectrophotometer (LANGE DR−6000, HACH, Germany) at a wavelength of 274 nm. The adsorption capacity and percentage of photocatalytic degradation were calculated using the following equation:

Adsorption capacity (qe) mg/g,

(1) $$q_{e}=\left(C_{0}-C_{e}\right)V/m$$

(2) $$\%\;\text{degradation}=\left(C_{0}-C\right)/C_{0}\times 100$$

Where, C_0_, C and C_e_ represent the initial concentration, concentration at reaction time and concentration at adsorption equilibrium (mg/L), V is the volume (L) of 2-CP solution and m (g) is the weight of the adsorbent.

The adsorption kinetic study was performed by fitting the obtained time-dependent data to pseudo-first order and pseudo-second order. The linear equations are represented as follows:

(3) $$\text{Pseudo}-\text{first}\;\text{order}\;\text{kinetic}:\;\text{log}\left(q_{e}-q_{t}\right)=\text{log}\;q_{e}-k_{1}t/2.303$$

(4) $$\text{Pseudo}-\text{second}\;\text{order}\;\text{kinetic}:\;t/q_{t}=1/k_{2}{q_{e}}^{2}+t/q_{e}$$

where q_e_ and q_t_ represent the adsorption capacity (mg/g) at equilibrium and time t (min), respectively. k_1_ is the pseudo-first order rate constant and k_2_ (g/min) is the pseudo-second order rate constant.

The rate of 2-CP photocatalytic degradation by C_3_N_4_ was also analyzed to investigate the photocatalytic behavior of the catalysts. The zero-order, first-order, second-order kinetic models were applied to analyze the experimental data at different contact times. The linear equations of zero-order, first-order, and second-order kinetic models are represented, respectively, as follows:

(5) $$\text{Zero}-\text{order}\;\text{kinetic}:\;C=C_{0}-k_{0}t$$

(6) $$\text{First}-\text{order}\;\text{kinetic}:\;\text{ln}\left(C/C_{0}\right)=-k_{1}t$$

(7) $$\text{Second}-\text{order}\;\text{kinetic}:\;1/C=\left(1/C_{0}\right)+k_{2}t$$

where C_0_ and C are 2-CP concentration at initial and reaction time t (min). k_0_ (mg/L. min), k_1_ (1/min) and k_2_ (L/mg.min) are zero-order, first-order and second-order rate constants, respectively.

## Declaration of Competing Interest

The authors declare that there is no conflict of interest.

## Appendix Supplementary Materials

Image, application 1
